# Usefulness of close surveillance for rectal cancer patients after neoadjuvant chemoradiotherapy

**DOI:** 10.1515/med-2022-0555

**Published:** 2022-09-05

**Authors:** Yu-Jen Hsu, Yih-Jong Chern, I-Li Lai, Sum-Fu Chiang, Chun-Kai Liao, Wen-Sy Tsai, Hsin-Yuan Hung, Pao-Shiu Hsieh, Chien-Yuh Yeh, Jy-Ming Chiang, Yen-Lin Yu, Jeng-Fu You

**Affiliations:** Division of Colon and Rectal Surgery, Chang Gung Memorial Hospital, Chang Gung University College of Medicine, Guei-Shan, Tao-Yuan, Taiwan; School of Traditional Chinese Medicine, Chang Gung University, Graduate Institute of Clinical Medical Sciences, Chang Gung University, Tao-Yuan, Taiwan; Division of Colon and Rectal Surgery, Chang Gung Memorial Hospital, Chang Gung University College of Medicine, Keelung, Taiwan; Division of Colon and Rectal Surgery, Chang Gung Memorial Hospital, Chang Gung University College of Medicine, Linko, 5, Fu-Hsing Street, Guei-Shan, Tao-Yuan, Taiwan

**Keywords:** rectal cancer, chemoradiotherapy, complete response, watch and wait, salvage surgery

## Abstract

It is controversial whether patients who achieve clinical complete remission (cCR) of rectal cancer should be treated with the “watch and wait” (W&W) or radical resection (RR) strategy. Our study aimed to compare the survival outcomes and ostomy rate of the W&W and RR strategies. Between January 2008 and December 2015, we investigated 26 patients who achieved pathologic complete remission after undergoing RR and 36 patients who adopted the W&W strategy because of cCR. The tumor regrowth, salvage surgery, recurrence, disease-free, and overall survival (OS) rates were assessed. In our study, recurrences occurred in nine and two patients from the W&W and RR groups, respectively. Each patient in the RR group had a temporary or permanent ostomy, but only three (8.3%) had an ostomy in the W&W group. The 5-year recurrence rate was 25.0% in the W&W group and 7.7% in the RR group. Six patients (16.7%) had tumor regrowth in the W&W group, and all were resectable when regrowth. The 5-year OS rates between the two groups were nonsignificant. There is no specific risk factor for recurrence and OS. Under close surveillance, the W&W group achieved similar OS to the RR group and benefited from a lower ostomy rate.

## 1 Introduction

Radical resection (RR), namely, abdominoperineal resection (APR) or low anterior resection (LAR) with total mesorectal excision, is the standard treatment for rectal cancer. However, RR may cause adverse effects, such as perioperative mortality, anastomotic leakage, sexual and urinary dysfunction, and permanent sphincter dysfunction [1,2,3,4]. Although the rate of APR is decreasing, there are still substantial patients with rectal cancer requiring temporary or permanent ostomies. Most patients are reluctant to undergo ostomy. Moreover, 50–90% of patients who undergo sphincter-sparing surgery have LAR syndrome, which presents as a deterioration in bowel function [3]. Furthermore, for patients with locally advanced rectal cancer, RR had negative effects on the ostomy rate, bowel function, and morbidity. Therefore, alternative treatment strategies are being investigated [4,5].

In cases of locally advanced rectal cancer, neoadjuvant or concurrent chemoradiotherapy (CCRT) is usually recommended for reducing local recurrence [6]. Furthermore, with the progress in neoadjuvant CCRT, some patients have favorable outcomes and more often sphincteric preservation because of improved downstaging. Earlier, downstaged patients were treated with curative RRs [5]. Complete remission after neoadjuvant CCRT indicates not only possible omitting surgery but also favorable prognostic factors, with less risk of local recurrence and distant metastasis [7,8]. For patients with clinical complete remission (cCR) statuses, the watch and wait (W&W) strategy might provide a safe alternative to RR. Although several promising studies have investigated this strategy, the results have been neither uniformly accepted nor tested in randomized trials [5,9,10]. The true safety of W&W remains unclear and requires investigation.

Our study retrospectively compared the survival outcomes between W&W and RR groups of patients with locally advanced rectal cancer presented with cCRs and pathological complete remission (pCRs) after neoadjuvant CCRT at a single tertiary medical center.

## 2 Materials and methods

### 2.1 Patients and variables

Detailed data of 839 patients who were diagnosed with rectal adenocarcinoma and received neoadjuvant radiotherapy between January 2008 and December 2015 were retrospectively retrieved from the Colorectal Section Tumor Registry at Chang Gung Memorial Hospital. This study was approved by the hospital’s Institutional Review Board. Clinical staging was determined by a multidisciplinary team using computed tomography (CT), magnetic resonance imaging (MRI), or positron emission tomography (PET). Of the 839 patients, 159 were excluded because of concurrent distant metastases at diagnosis. Of the remaining 680 patients, 264 received short-course radiotherapies and immediate operations, but 43 patients did not complete the radiotherapy because of side effects (<4,500 cGy), and 62 (16.6%) achieved either cCR or pCR. cCR was defined as no suspicious residual mass in the bowel wall, which was visualized as a scar or only minor erosion under endoscopy but biopsy presented negative for malignancy; no enlarged extraluminal residual tumor or regional lymph nodes (LNs) detected in radiologic imaging, such as CT or MRI; no palpable tumor identified by clinical physician’s digital rectal examination during a period of 6–10 weeks after neoadjuvant chemoradiotherapy. In this institution, patients with rectal cancer are referred to surgical resection after neoadjuvant chemoradiotherapy, unless the patients refuse. The W&W strategy was adopted for patients who had cCR and refused to undergo surgical resection. pCR was defined as no pathologically defined residual viable cancer cells, either in the main tumor or in regional LNs, after patients underwent a rectal RR.

The available medical records comprised data on age, sex, tumor location (distance from the anal verge), maximal tumor diameter, carcinoembryonic antigen (CEA), clinical T and N stages, radiation dosage, neoadjuvant chemotherapy regimen, adjuvant chemotherapy regimen, morbidity in RR, and ostomy status. Two neoadjuvant radiotherapy regimens were implemented: long-course radiotherapy (5,040 cGy delivered in 28 fractions) and short-course radiotherapy (2,500 cGy delivered in five fractions). Concurrent chemotherapy was administered essentially by 5-Fluorouracil (5-Fu) or 5-Fu plus oxaliplatin.

Different physicians of the same department at this institution adopted similar follow-up routines. The patients were subjected to a follow-up program that comprised outpatient visits every 3 months with physical examinations, namely digital rectal exams and CEA tests, CT or MRI scans, and colonoscopies, at the physician’s discretion. Recurrent disease was confirmed through histology of colonoscopy biopsy specimens, reoperation, or radiological studies. Regrowth was defined as a tumor growing at the former site and could be easily detected through colonoscopy or digital examination. Prognoses were evaluated based on a 5-year recurrence rate and 5-year overall survival (OS). The interval of recurrence was defined as the duration between the date of finishing radiotherapy and the date of confirmation of recurrence. The OS interval was defined as the duration between the dates of finishing radiotherapy and death.

### 2.2 Statistical analysis

All analyses were performed using the Statistical Package for the Social Sciences, version 24.0 (IBM SPSS Statistics v.24). Clinicopathologic characteristics were compared using a chi-square test for categorical variables and Student’s *t* tests for continuous data. Recurrence, OS, and time-to-event probabilities were computed using univariate analyses applying the Kaplan–Meier method. Differences were estimated using the log-rank test. Statistical significance was set at *p* < 0.05. We examined the risk factors for recurrence and OS including W&W strategy, age ≥65, sex, high CEA level (≥5 ng/mL), neoadjuvant chemotherapy, the tumor, lymph node, metastasis (TNM) staging system and adjuvant chemotherapy with Kaplan–Meier survival analysis. If the “*p*-value <0.2” was observed from the Log rank test, then we applied the risk factor to the COX regression model. A univariate COX regression model was applied followed by a multivariate COX regression model backward stepwise (Wald) that was used to provide an estimate of the hazard ratio (HR) and its confidence interval (CI) for investigating the association between the survival time of patients and one or more predictor variables/factors.

## 3 Results

We enrolled 26 patients who received RR for rectal cancer with a pathologic result of ypT0N0M0 and 36 patients who completed the W&W strategy as a rectal cancer treatment and clinically achieved CR. The mean patient age was 58.5 years. After a median follow-up duration of 80.7 months, 11 patients had tumor recurrence. The overall recurrence rate of the 62 patients was 17.7%.

The demographic data of these patients are presented in Table 1. The W&W group had significantly older adult patients than did the RR group (age ≥65 years, 38.9% vs 7.7%, and *p* = 0.006). The tumor locations in the W&W group showed a closer distance to the anus than those in the RR group (W&W vs RR, 3.53 ± 1.76 cm vs 5.47 ± 2.30 cm, and *p* < 0.001). The pretreatment clinical T stages did not differ between the two groups, but the pretreatment clinical N stage was more advanced in the RR group than in the W&W group. The rate of receiving adjuvant chemotherapy was significantly higher in the W&W group than in the RR group (75% vs 30.8% *p* < 0.001). Each patient who received a RR had a temporary or permanent ostomy, but in the W&W group, only two patients had temporary ostomies and one had a permanent ostomy (overall ostomy rate, W&W vs RR, 8.3% vs 100%, and *p* < 0.001). Ten patients (38.5%) in the RR group experienced early and late morbidity, namely, anastomosis leakage, wound infection, bladder dysfunction, abdominal abscess, and bowel obstruction. The overall recurrence rate was higher in the W&W group (25.0%) than in the RR group (7.7%), although this difference was nonsignificant (*p* = 0.099).

**Table 1:** Demographic data

	Watch & wait	Radical resection	*p*-Value
	*N* = 36	*N* = 26	
	Number (%)	Number (%)	
Sex (male)	25 (69.4)	20 (76.9)	0.515
Age ≥65 years	14 (38.9)	2 (7.7)	0.006
Tumor location (distance from anal verge, cm)	3.53 ± 1.76	5.47 ± 2.30	<0.001
CEA ≥ 5	4 (10.8)	2 (7.7)	0.653
Clinical T stage			0.486
T1	2 (5.6)	0 (0)	
T2	5 (13.9)	3 (11.5)	
T3	28 (77.8)	23 (88.5)	
T4	1 (2.8)	0 (0)	
Clinical N stage			0.001
N0	29 (80.6)	9 (34.6)	
N1	7 (19.4)	11 (42.3)	
N2	0 (0)	6 (23.1)	
Dosage of RT (cGy)			0.667
5,040 (long course)	34 (94.4)	25 (96.2)	
2,500 (short course)	1 (2.8)	0 (0)	
Incomplete RT course	1^a^ (2.8)	1 + (3.8)	
Neoadjuvant chemotherapy			0.370
5-FU/LV	32 (88.9)	21 (80.8)	
FOLFOX6	4 (11.1)	5 (19.2)	
Adjuvant chemotherapy (5-FU/LV)			<0.001
No	9 (25.0)	18 (69.2)	
<6 months	21 (58.3)	2 (7.7)	
≥6	6 (16.7)	6 (23.1)	
Ostomy status			<0.001
No	32 (88.9)	0 (0)	
Temporary	2 (5.6)	23 (88.5)	
Permanent	2 (5.6)	3 (11.5)	
Morbidity	0	10(38.5)	
Median follow-up time (months)	74.0	116.5	
Recurrence	9 (25.0)	2 (7.7)	0.099

CEA, carcinoembryonic antigen; cGy, centigray; RT, radiotherapy.

^a^4,860 cGy; ^+^3,780 cGy.

Recurrences were observed in eleven patients during follow-up (nine from the W&W group and two from the RR group), and the profile of these patients is shown in Table 2. Two patients from the RR group experienced recurrences within 1.5 years: one patient had a local recurrence (6.8 months after the operation) followed by multiple metastases and death 2.3 years after the operation, and one patient had lung metastasis 16.5 months after the operation and died 6.1 years after the operation. Six patients in the W&W group had tumor regrowth at the previous tumor site within 3 years; selected colonoscopy findings are shown in Figure 5. Four of these six patients underwent salvage surgery. Of these, three had no recurrences after salvage surgery, whereas one patient had a second episode of local recurrence at the time of follow-up. Three patients were unresectable when recurrence in the W&W group. One patient had a first recurrence of liver metastasis at 14.7 months after completing CCRT. One patient exhibited a presacral recurrence through CT during an elevated CEA examination 2.5 years after treatment. The third patient was recurrent at 58 months after complete CCRT because of PA node and lung metastasis.

**Table 2:** Recurrence data

Patient No.	Group	Age	Tumor location (distance from anal verge, cm)	Location of first recurrence	Recurrence time (months)	Salvage surgery	Status after recurrence
1	W&W	57	3	Regrowth	22.3	No	Refused salvage surgery
2	W&W	49	2	Regrowth	13.6	LAR	Recurrence again after operation
3	W&W	54	1	Regrowth	13.6	APR	Disease free
4	W&W	70	2	Regrowth	18.8	APR	Disease free
5	W&W	57	5	Regrowth	23.2	LAR	Disease free
6	W&W	61	2	Pelvic^a^	29.4	No	Chemotherapy
7	W&W	72	5	Liver	14.7	No	Chemotherapy
8	W&W	78	3	Lung, PA node	57.92	No	Chemotherapy
9	W&W	60	3	Regrowth	31.11	No	Escape
10	RR	52	5	Pelvic	8.4	—	Multiple metastasis
11	RR	49	3	Lung	19.2	—	Multiple metastasis

W&W, watch and wait; LAR, low anterior resection; APR, abdominoperineal resection; and RR, radical resection.

^a^Presacral region.

**Figure 1:**
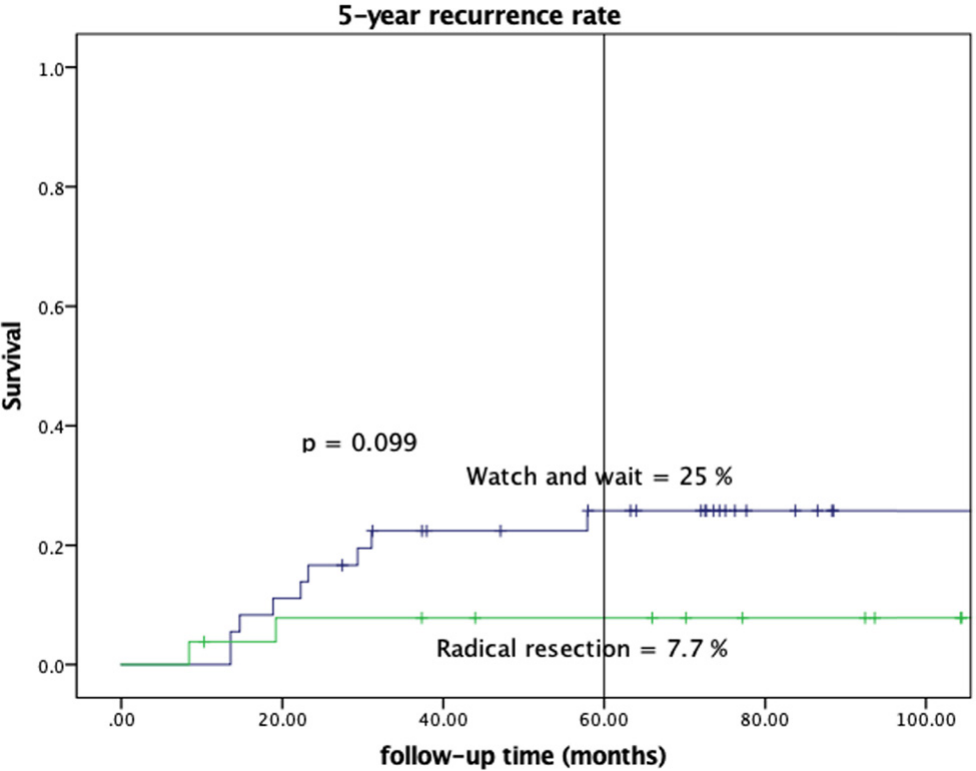
Local recurrence rate.

**Figure 2:**
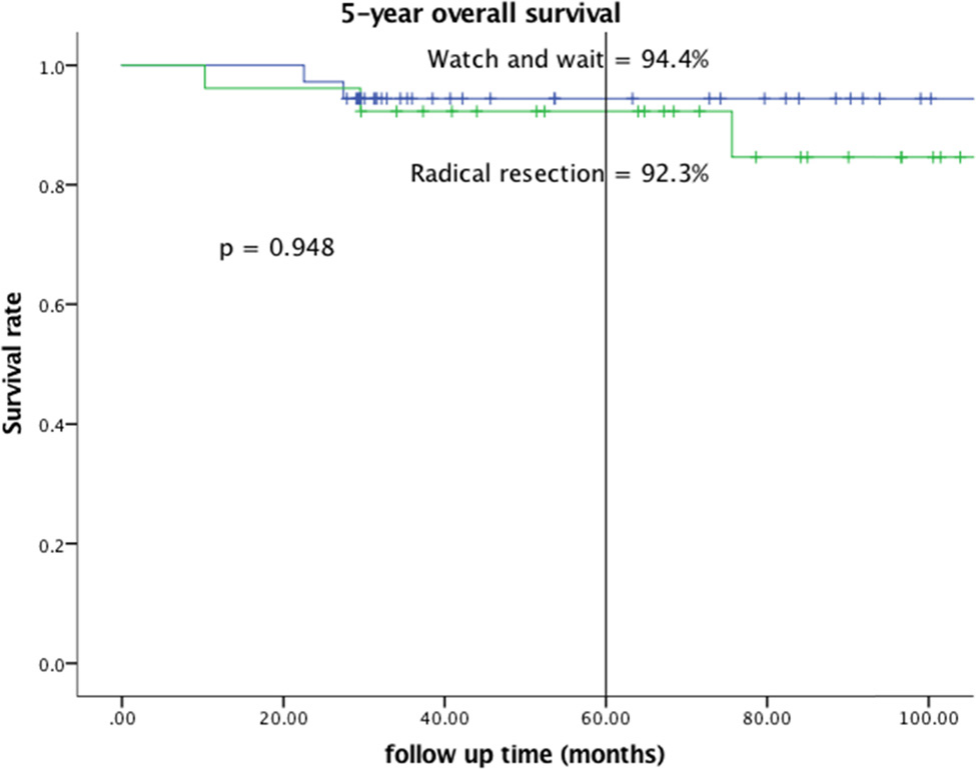
Overall survival.

The mean follow-up time was 77.9 months in the W&W group and 103.0 months in the RR group. The estimated 5-year recurrence rate was 25.0% in the W&W group and 7.7% in the RR group, but the difference was nonsignificant (*p* = 0.099). The estimated 3-year DFS rate was 77.8% in the W&W group and 88.5% in the RR group. The log-rank test result comparing 3-year DFS curves between the W&W and RR groups was nonsignificant (Figure 1, *p* = 0.126). The estimated 5-year OS rate was 94.4% in the W&W group and 92.3% in the RR group, and this difference was nonsignificant (Figure 2, *p* = 0.948).

After univariable COX regression, we selected these factors below 0.2 for multivariable COX regression. W&W strategy, age ≥ 65, sex, high CEA level (≥5 ng/mL), neoadjuvant chemotherapy, TNM stage, and adjuvant chemotherapy were all not independent risk factors for recurrence nor OS. All adjusted parameters, HR along with 95% CI and *p*-value, are listed in Tables 3 and 4.

**Table 3:** Cox regression analysis of risk factors for recurrence

	Univariate		Multivariate	
	HR (CI-95%)	*p*-Value	HR (CI-95%)	*p*-Value
W&W	3.361 (0.725–15.57)	0.121	2.046 (0.389–10.77)	0.398
Age ≥65	1.173 (0.311–4.423)	0.814		
Male	4.252 (0.544–33.23)	0.168	3.653 (0.457–29.19)	0.222
Stage II	1.064 (0.285–3.965)	0.926		
Stage III	0.161 (0.018–1.439)	0.102	1.130 (0.295–4.328)	0.858
cT3/4 (compare cT1/2)	0.514 (0.149–1.778)	0.293		
N1	0.200 (0.025–1.584)	0.128	0.246 (0.024–2.545)	0.239
N2	0.718(0.091–5.671)	0.753		
CEA ≥5	0.929 (0.119–7.264)	0.944		
NCT = 5-FU/LV	—			
NCT = FOLFOX6	0.038 (0.000–38.15)	0.353		
ACT < 6 months	1.376 (0.369–5.126)	0.634		
ACT ≥ 6 months	1.055 (0.193–5.762)	0.951		

**Table 4:** Cox regression analysis of risk factors for OS

	Univariate		Multivariate	
	HR (CI-95%)	*p*-Value	HR (CI-95%)	*p*-Value
Warch and Wait	1.261 (0.277–5.745)	0.764		
Age ≥65	3.043 (0.661–14.01)	0.153	3.549 (0.768–16.40)	0.105
Male	0.473 (0.055–4.075)	0.495		
Stage II	0.974 (0.216–4.382)	0.972		
Stage III	N/A	0.964		
cT3/4(compare cT1/2)	0.613 (0.136–2.762)	0.524		
N1	N/A	0.967		
N2	0.956 (0.115–7.955)	0.967		
CEA ≥5	1.504 (0.181–12.53)	0.706		
NCT = 5-FU/LV	—			
NCT = FOLFOX6	0.037 (0.000–176.9)	0.445		
ACT < 6 months	0.246 (0.029–2.076)	0.197	0.207 (0.024–1.772)	0.150
ACT ≥ 6 months	N/A	0.974		

NCT, neoadjuvant chemotherapy; ACT, adjuvant chemotherapy.

## 4 Discussion

In this study, we analyzed patients who received neoadjuvant CCRT for rectal cancer and achieved cCR (W&W group) or pCR (RR group). Patients in the W&W group were significantly older than those in the RR group. The tumor location in the W&W group was closer to the anus than that in the RR group. Moreover, patients in the W&W group were more frequently ostomy free than those in the RR group. However, no significant differences were observed in the 5-year recurrence rate, 3-year DFS, and 5-year OS rates between the two groups.

In the literature review, the oncologic outcomes were good and similar in patients with cCR who underwent the W&W strategy or RR. Habr-Gama, a pioneer in the W&W policy, compared the outcomes of patients with an incomplete clinical response and pathologically confirmed pT0N0 stage after surgery with those who applied the W&W strategy due to assume a complete clinical response. The 5-year OS and DFS rates were 100 and 92% in a W&W group and 88 and 83% in a RR group, respectively [11]. Other studies from the Netherlands, United States, and United Kingdom also reported similar DFS and OS between W&W and control groups. [10,12,13,14,15] In our study, the 5-year OS and 3-year DFS rates were 94.4 and 77.8%, respectively, in the W&W group and 92.3 and 88.5%, respectively, in the RR group, with no significant differences. This result is similar to that of Habr-Gama, recently adopted in the world.

The oncologic outcomes that we observed were comparable between the W&W strategy and pCR after radical surgery, but the functional outcomes differed. Patients who did not undergo radical surgery had better functional outcomes than patients who underwent radical surgery. Maas et al. and Pucciarelli et al., respectively, reported that up to 78 and 65% of patients still had bowel function problems 1 year after CCRT and RR [14,16]. Many studies have also mentioned the high colostomy and complication rates after CCRT and radical surgery. Maas et al. reported that 45% of patients required a permanent colostomy and 55% required a temporary colostomy. Major and minor complications, namely anastomosis leakage, intra-abdominal abscess, urinary retention, and wound infection, were reported in 50% of patients who underwent radical surgery [14]. The complication rate was also very high after RR in our study. Eight patients (30.8%) experienced early morbidity and two (7.7%) late morbidity, namely anastomosis leakage, wound infection, bladder dysfunction, abdominal abscess, and bowel obstruction. Renehan et al. reported that patients in a W&W group had significantly better 3-year colostomy-free survival than those in a RR group (74% vs 47%) [17]. In our study, the colostomy rate was significantly lower in the W&W group than in the RR group. Six patients in this group experienced tumor regrowth within 3 years of the W&W strategy, and four patients underwent salvage surgery. Two of these four patients had permanent colostomies and two had temporary colostomies. In the RR group, three patients had permanent colostomies (11.5%) and 23 (88.5%) had temporary colostomies. All 23 patients underwent colostomy closures within 1 year, but three (11.5%) experienced delayed anastomosis complications and underwent another diverted colostomy. The high surgical risk and the high colostomy rate in the RR group have explained why most elders preferred to choose W&W rather than surgery.

**Figure 3:**
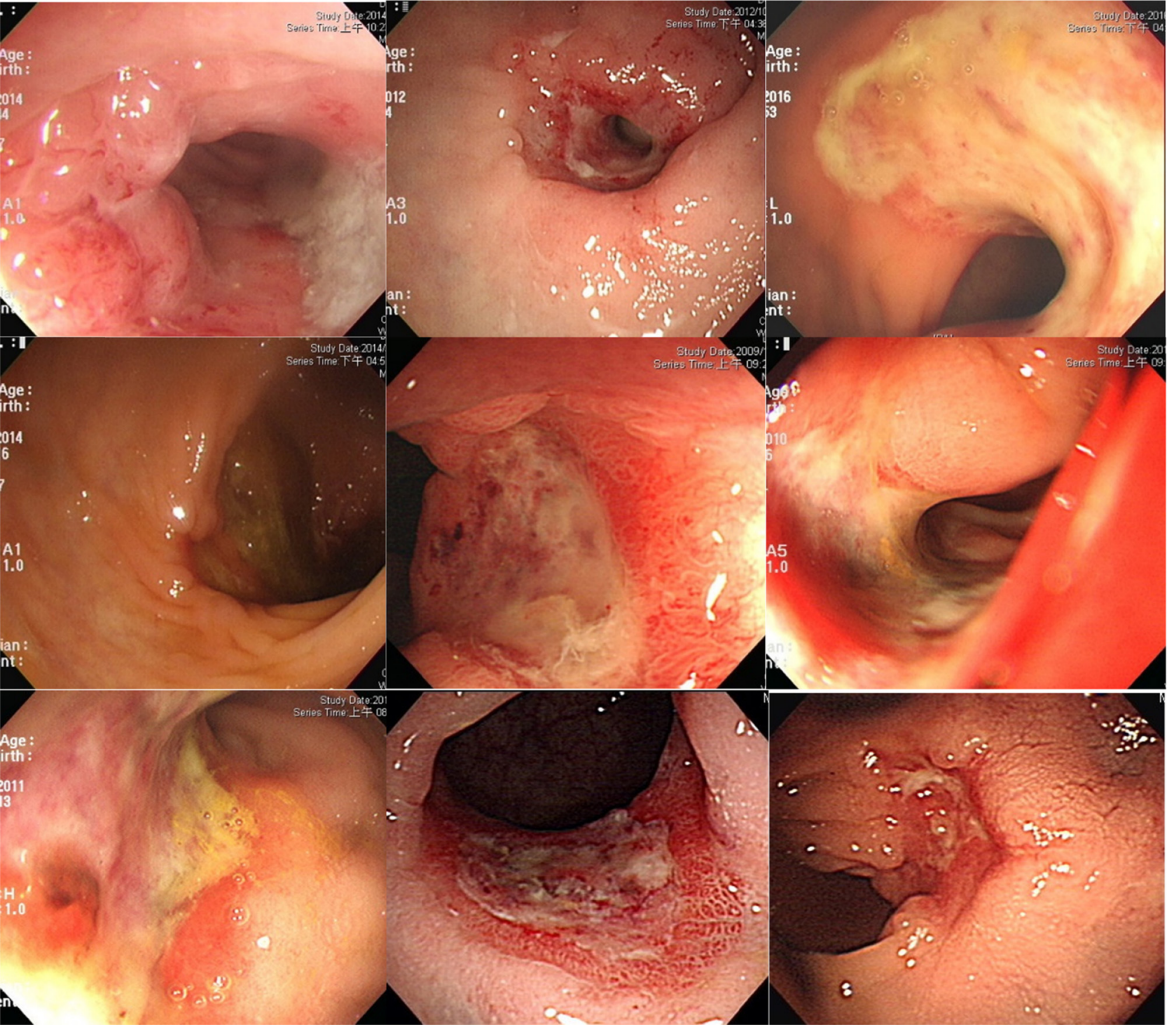
Colonoscopy findings of patients in the RR group who did not achieve cCR.

In our institution, for patients with rectal cancer who achieve cCR after neoadjuvant CCRT, both W&W and RR strategies are considered. The final choice of W&W or RR is determined based on patient willingness and physician discretion. Nevertheless, we prefer RR because of the difficulty of mesorectal LN detection through imaging modalities. cCRs were evaluated through digital examination, colonoscopy, CEA, and imaging modalities (CT, MRI, or PET). Digital examination played a crucial role in our data analysis. The patients with lower tumor location (within 5 cm from the anal verge) may have preferred the W&W strategy over a permanent ostomy. It is relatively easy to evaluate the tumor status in the lower rectal tumors through digital examination. The higher tumor location in the RR group made it relatively difficult to obtain information objectively from digital examinations. Therefore, we determined the presence of residual tumors through sigmoidoscopy and imaging modalities (CT/MRI/PET). Sigmoidoscopy images of the RR group showed that many of the tumors did not achieve cCRs. At least nine images showed erosion and ulceration without white scar formation (Figure 3). In the RR group, other than the relatively long distances from the anal verge, which make it difficult to evaluate the tumor through digital examination, the detection of positive LNs through preradiotherapy imaging modalities may also guide the choice of RR. Although the accuracy of imaging modalities (CT/MRI/PET) does not enable precise determination of LN status, [18,19] clinical physicians would not risk the possibility of LN metastases and would therefore choose the RR strategy. Conversely, a pooled analysis reported a 5–10% incidence of positive LNs in ypT0 patients [7,20]. Our physicians adopted relatively strict selection criteria for the W&W group to prevent regional LN metastases. However, one patient still had a tumor recurrent in the presacral region at 29.4 months after radiotherapy. The patient was detected of tumor recurrence according to CEA elevation and a CT image was proved. The sigmoidoscopy examination revealed no tumor regrowth in this patient.

**Figure 4:**
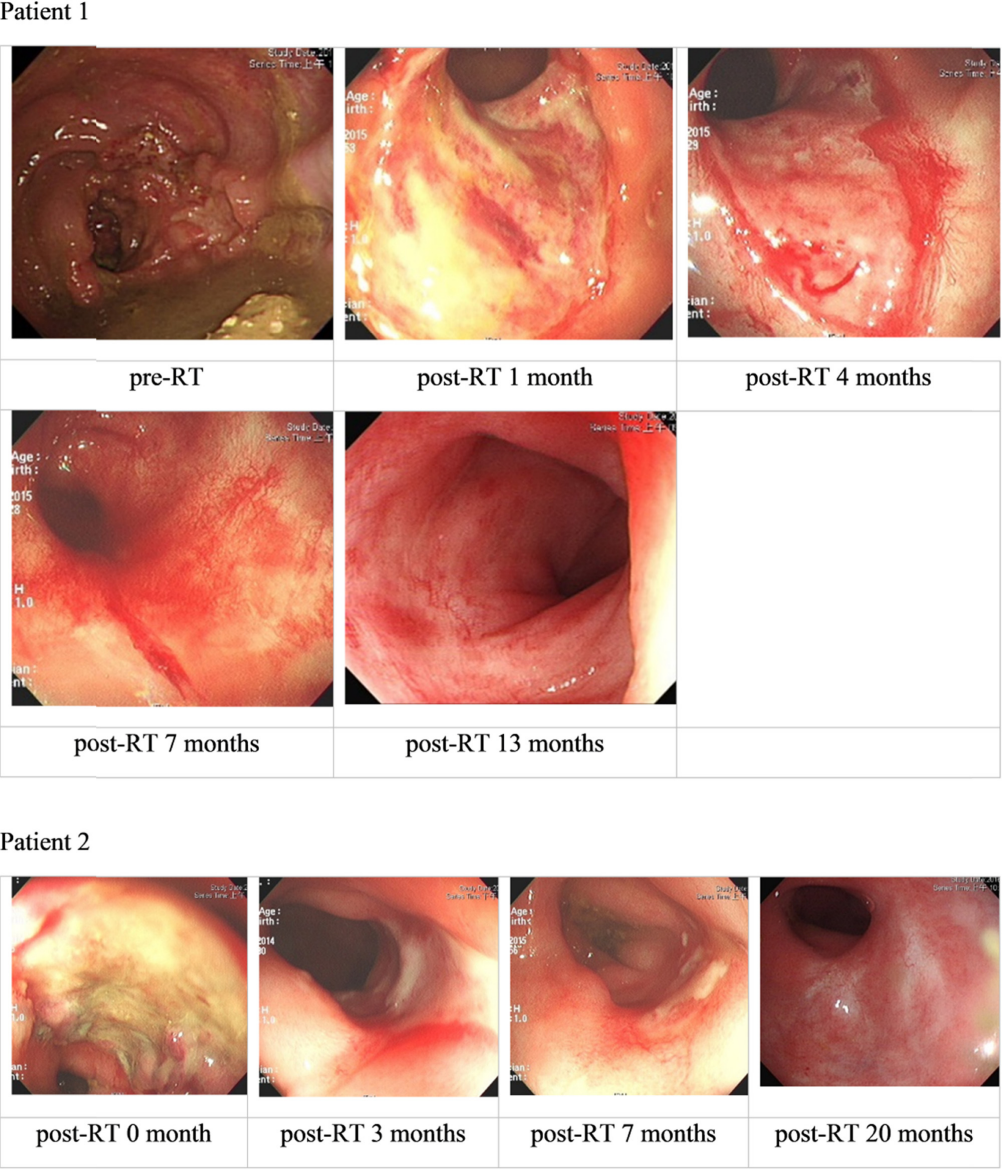
Patients of the W&W group with delayed white scar formations. Patient 1: Tumor was located 5 cm from the AV. Long-course RT with uracil-tegafur had delayed scar formation for approximately 1 year. No tumor regrowth occurred for 68.5 months after radiotherapy, and the patient is alive. Patient 2: The tumor was located 5 cm from the AV. Long-course RT with Xeloda had delayed white scar formation for >7 months. No tumor regrowth occurred for 77.0 months after radiotherapy, and the patient is alive.

**Figure 5:**
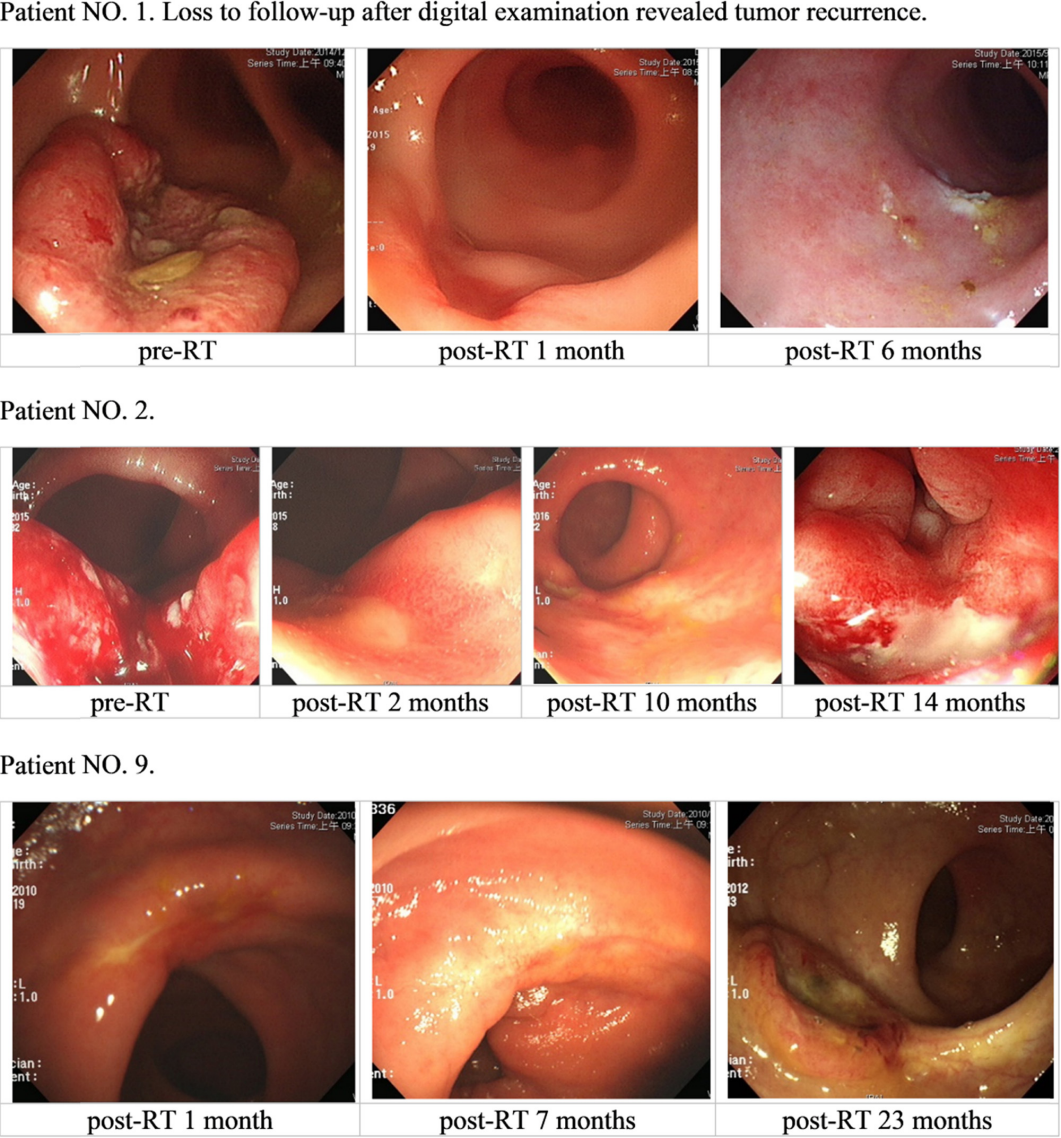
Colonoscopy findings in patients with tumor regrowth after applying the W&W strategy.

There are some tips according to our experience in the study. First, in patients in the W&W group, two of 36 patients presented delayed white scar formation with an ulcerative or erosive lesion over the previous tumor area that persisted for more than 6 months, which very slowly transitioned to a scar (Figure 4). Second, the digital examination revealed soft instead of fixed or induration lesions, which favored continuing the W&W strategy instead of opting for surgical intervention. Third, patients with sigmoidoscopy images showing a white scar have to be monitored every 3 months for 3 years. The proportion of patients with cCR who develop tumor regrowth after the W&W strategy varies from 5% to 60% [4,10,14,15,21,22,23]. In our study, six patients (16.7%) experienced tumor regrowth within 3 years in the W&W group (Figure 5). Four of these patients underwent salvage surgery after tumor regrowth. One refused an operation because of end-stage renal disease comorbidity and cardiovascular disease. The other one escaped for an unknown reason. Of these patients, three remained tumor-free during the follow-up after the salvage surgery, and one patient had a local recurrence 7 months after the operation.

pCR is defined as the absence of cancer cells in a surgical site after neoadjuvant CCRT and resection. Moreover, 15–40% of patients who receive neoadjuvant CCRT will achieve a pCR [7,21,24]. The interval between CCRT completion and operation time affects pCR rates. Many trials have reported that with total neoadjuvant therapy, some patients were eligible for organ preservation due to higher frequencies of CR rates [8]. This may result from more preoperative systemic chemotherapy and a prolonged interval between CCRT completion and the operation. In our study, the cCR and pCR rates were 16.6%, which is a lower standard in other studies. In our study, more than half of the patients underwent RR within 8 weeks after CCRT completion. Our physicians strictly complied with the requirements of the W&W group and often performed RR to avoid distant or regional metastases during observation after CCRT completion. Furthermore, to avoid imprecise detection of cCR, more patients received 5-FU-based adjuvant chemotherapy in the W&W group than in the RR group.

### 4.1 Limitations

Our study has several limitations. First, this study is retrospective with a limited sample size, which can cause various biases. Second, patient selection for the W&W group was nonstandardized and varied according to the physicians, and patients were mostly assigned based on CTs/MRIs, colonoscopies, and digital rectal examinations. In this study, the CR rate was 16.6%, which is lower than that in other studies and indicates that the physicians were careful when adopting the W&W strategy, which significantly affected the comparisons between the W&W and RR groups. It is unlikely that a randomized trial of W&W versus RR for cCR after neoadjuvant CCRT will be logistically and ethically feasible. The combined experiences of institutions using the W&W method in cCR may help promote it as a safe treatment strategy.

## 5 Conclusions

In conclusion, patients with rectal cancer who receive CCRT and achieve cCR may avoid RR or ostomy through close surveillance, including CEA tests, CT, MRI, colonoscopies, and digital rectal examinations. In our results, patients in the W&W group were more frequently ostomy free than those in the RR group. Also, no significant differences were observed in the 5-year recurrence rate and 5-year OS rates. Although tumor regrowth was noted at previous tumor sites in the W&W group, many of the patients who received salvage surgery became disease-free. Because this is a retrospective study with some possible decision factors like the patient’s age, tumor location, and clinical N stage, for “W&W” or “RR,” the safety of the W&W strategy needs to be confirmed by setting a standardized cCR definition with further multi-institutional and randomized controlled trials.
